# Four new species of *Metapocyrtus* Heller, 1912 (Coleoptera, Curculionidae, Entiminae, Pachyrhynchini) from Mindanao Island, Philippines

**DOI:** 10.3897/BDJ.9.e72453

**Published:** 2021-09-28

**Authors:** Analyn Anzano Cabras, Milton Norman Medina

**Affiliations:** 1 University of Mindanao, Davao City, Philippines University of Mindanao Davao City Philippines

**Keywords:** Davao de Oro, mimicry, Mindanao Island, Pachyrhynchini, urban biodiversity, weevils

## Abstract

**Background:**

The genus *Metapocyrtus* Heller, 1912 is the most speciose and complex amongst the tribe Pachyrhynchini with seven subgenera and more than 200 described species. The genus is endemic to the Philippines and remains largely unknown particularly in the less explored areas or mountains.

**New information:**

Four new species of *Metapocyrtus* Heller, 1912 (Coleoptera, Curculionidae, Entiminae, Pachyrhynchini), are described from Mindanao Island, Philippines. Brief bionomical notes and mimicry with their sympatric beetles and other insect counterparts are also reported.

## Introduction

The genus *Metapocyrtus* Heller 1912, belonging to the tribe Pachyrhynchini Schönherr 1826, is the most diverse and complex endemic member of the tribe, with more than 200 described species. The tribe Pachyrhynchini contains 18 genera with more than 500 described species distributed from the Philippines to Papua New Guinea, Australia, Taiwan, Japan and Indonesia (Schönherr 1826, Alonso-Zarazaga and Lyal 1999) and has its centre of diversity in the Philippines. The general diagnostic characters of this tribe include having mandibles without a scar or lasting appendage on the exterior surface, evenly arcuate at sides; antennal scrobes lateral, curving downwards in front of eyes at sides of rostrum; elytra with rounded humeri; hind coxae broadly contiguous with elytra at sides (Morimoto et al. 2006). *Metapocyrtus* is easily distinguished from other pachyrhynchines by the following characteristics: “Rostrum apically not swollen, basally with a more or less strongly pronounced transverse groove; scape of antenna reaching at least to or beyond hind margin of eye.” (Schultze 1925, p. 135). Currently, the genus is divided into seven subgenera (i.e. *Artapocyrtus* Heller, *Dolichocephalocyrtus* Schultze, *Metapocyrtus* Heller, s. str., *Orthocyrtus* Heller, *Sclerocyrtus* Heller, *Sphenomorphoidea* Heller and *Trachycyrtus* Heller), based on the combined characteristics of the rostrum, pronotum and body (Schultze 1925, Yap and Gapud 2007). Studies about this taxon have increased recently with several species descriptions done by both local and foreign workers (Cabras et al. 2018, Cabras et al. 2019, Bollino et al. 2020, Cabras et al. 2021). Despite the large advancement of the studies of this genus in the past years, the taxonomic classification of this group is still chaotic, with many ill-defined subgenera and species groups which need thorough review.

In our examination of the specimens at the Coleoptera Research Center, four species were found to be novel to science. Three of the new species are tentatively assigned under subgenusMetapocyrtus Heller, based on the following characters elucidated by Yap and Gapud (2007): a) head and rostrum short, 0.75 to 0.85 times as long as wide, b) rostrum with a V-shaped ridge forming a shallow triangular depression, c) rostrum with rounded dorsolateral edges, d) anterior margin of pronotum truncate and strongly pronounced and e) elytra laterally elliptical or dorsally ovate. Meanwhile, one of the new species is not assigned any subgeneric classification. In this paper, the four new species are described with brief notes on their ecology and mimetic relationships with other weevils and insects. Due to the little or almost no use of the female genitalia in identifying and characterising the different species of Pachyrhynchini (Bollino et al. 2020), we avoided illustrating the said anatomical parts.

## Materials and methods

Prior to collection, Gratuitous Permits were obtained from the Department of Environment and Natural Resources. For collection, the specimens deposited in the University of Mindanao Coleoptera Research Center were collected through sheet beating and hand picking and killed in vials with ethyl acetate. Morphological characters were observed under Luxeo 4D and Nikon SMZ745T stereomicroscopes. The illustrations, as well as the treatment of the genitals, were identical to those described by Yoshitake (2011). Images of the habitus and genitalia were taken, using a Nikon D5300 digital camera with a Sigma 18–250 macro lens. All images were stacked and processed using a licensed version of Helicon Focus 6.7.0 and Photoshop CS6 Portable software. Label data are indicated verbatim. Measurements and labels mentioned in this paper are abbreviated as follows:

/ indicates different lines of text in a label

// indicates different labels on the same specimen.

â arithmetic mean rounded to one decimal place;

LB body length, from the apical margin of pronotum to the apex of elytra;

LE elytral length, from the level of the basal margins to the apex of elytra;

LP pronotal length, from base to apex along the mid-line;

LR length of rostrum;

WR maximum width across the rostrum;

WE maximum width across the elytra;

WP maximum width across the pronotum.

Comparative materials and specimens, used in the study, are deposited in the following institutional collections:

**DUBC** Daugavpils University Beetle Collection, Daugavpils, Latvia;

**SMTD** Senckenberg Natural History Collections, Dresden, Germany;

**UMCRC** University of Mindanao Coleoptera Research Center, Davao City, Philippines.

## Taxon treatments

### Metapocyrtus jumawani

Cabras & Medina, 2021 sp. n.

E3BD3795-7D06-5AA8-AC1E-ABA1506AD9F1

urn:lsid:zoobank.org:act:667bb1ca-a668-44f9-8c00-c1c21c7ddaf8

#### Materials

- **Type status:** Holotype. **Occurrence:** recordedBy: Local collector; individualCount: 1; sex: male; lifeStage: adult; preparations: card-mounted; disposition: in collection; **Taxon:** scientificName: *Metapocyrtusjumawani*; kingdom: Animalia; phylum: Arthropoda; class: Insecta; order: Coleoptera; family: Curculionidae; genus: Metapocyrtus; specificEpithet: *jumawani*; taxonRank: species; scientificNameAuthorship: Cabras & Medina, 2021; nomenclaturalCode: ICZN; **Location:** continent: Asia; islandGroup: Mindanao; country: Philippines; countryCode: PHL; stateProvince: Davao de Oro; municipality: Maragusan; locality: Langgawisan; **Identification:** identifiedBy: AA Cabras; **Event:** samplingProtocol: handpicking; year: 2018; month: March; habitat: riparian; **Record Level:** institutionID: UM; collectionID: UM-CRC
- **Type status:** Paratype. **Occurrence:** recordedBy: Local collector; individualCount: 2; sex: male; lifeStage: adult; preparations: card-mounted; disposition: in collection; **Taxon:** scientificName: *Metapocyrtusjumawani*; kingdom: Animalia; phylum: Arthropoda; class: Insecta; order: Coleoptera; family: Curculionidae; genus: Metapocyrtus; specificEpithet: *jumawani*; taxonRank: species; scientificNameAuthorship: Cabras & Medina, 2021; nomenclaturalCode: ICZN; **Location:** continent: Asia; islandGroup: Mindanao; country: Philippines; countryCode: PHL; stateProvince: Davao de Oro; municipality: Maragusan; locality: Langgawisan; **Identification:** identifiedBy: AA Cabras; **Event:** samplingProtocol: handpicking; year: 2018; month: March; habitat: riparian; **Record Level:** institutionID: UM; collectionID: UM-CRC
- **Type status:** Paratype. **Occurrence:** recordedBy: Local collector; individualCount: 4; sex: female; lifeStage: adult; preparations: card-mounted; disposition: in collection; **Taxon:** scientificName: Metapocyrtusjumawani; kingdom: Animalia; phylum: Arthropoda; class: Insecta; order: Coleoptera; family: Curculionidae; genus: Metapocyrtus; specificEpithet: jumawani; taxonRank: species; scientificNameAuthorship: Cabras & Medina, 2021; nomenclaturalCode: ICZN; **Location:** continent: Asia; islandGroup: Mindanao; country: Philippines; countryCode: PHL; stateProvince: Davao de Oro; municipality: Maragusan; locality: Langgawisan; **Identification:** identifiedBy: AA Cabras; **Event:** samplingProtocol: beating sheet; year: 2018; month: March; habitat: riparian; **Record Level:** institutionID: UM; collectionID: UM-CRC

#### Description

**Male.** Dimensions (in mm): N = 5. LB 7.4–8.9 (holotype 8.2, â: 8.5), LR 1.1–2.0 (2.0, â: 1.6), WR 1.1–1.2 (1.2, â: 1.2), LP 2.8–3.2 (3.2, â: 3.0), WP 2.8–3.4 (3.4, â: 3.0), LE 4.6–5.7 (5.0, â: 5.5), WE 2.3–4.5 (4.0, â: 3.7).

**Figure 1.**
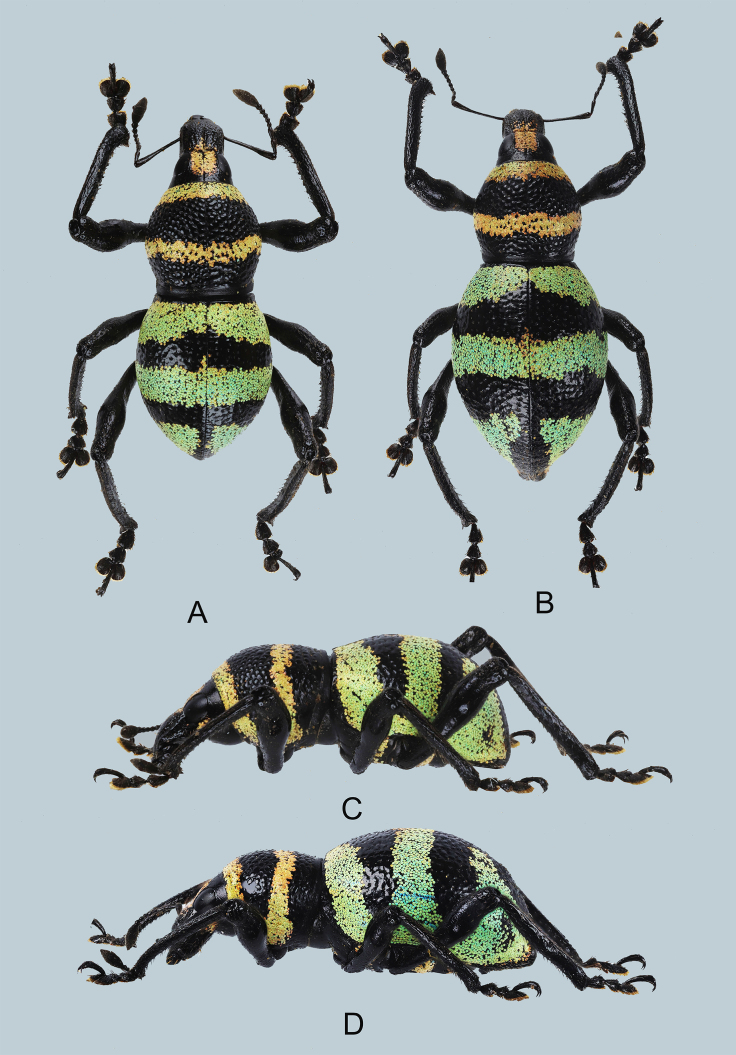
***Metapocyrtusjumawani*** sp. nov.: **A** male holotype, dorsal view; **B** female, dorsal view; **C** male, lateral view; **D** female, lateral view.

Habitus as shown in Fig. 1A–D.

Integument black. Body surface, rostrum, head and underside weakly lustrous.

Body subglabrous. Head subglabrous with sparse and minute pubescence; forehead between eyes slightly depressed, covered with metallic, golden-yellow, round scales; lateral parts with elongated patch of golden-yellow round scales below the eyes and metallic golden-yellow piliform scales towards ventral side; median groove distinct. Ros­trum weakly rugose with sparse, minute pubescence, slightly longer than wide (LR/WR 1.66), dorsum bears minute, brownish hairs; baso-lateral surface with golden-yellow piliform scales which become longer towards anterolateral surface; apex with long, yellowish hairs; transverse basal groove distinct; dorsal surface convex. Eyes medium-sized and feebly convex. Antenna moderately clavate, scape slightly shorter than funicle, moderately covered with fine, long, recumbent, brownish hairs. Funicular antennomeres I and II almost of the same length, nearly three times longer than wide; antennomeres III–VII slightly longer than wide; club subovoid, nearly three times longer than wide. Prothorax subglobular, as long as wide (LP/WP 1.0), coarsely granulated with sparse minute pubescence, widest at mid-length, weakly convex, with a faint groove along midline reaching only mid-length and with the following scaly markings of metallic golden-yellow, round scales: a) thin band approximately 1/6 length of pronotum along apical margin, b) transverse band across entire width just slightly behind mid-length and c) lateroventral stripe before the coxa, confluent with anterior marginal band and transverse band at mid-length. Elytra short, subovate (LE/WE 1.25), wider and longer than prothorax (WE/WP 1.18, LE/LP 1.56), subglabrous, weakly convex, coarsely punctured, each puncture with light-coloured, short seta; each elytron with the following scaly markings; a) broad basal band nearly twice as broad as pronotal bands extending from suture to lateral margin, b) broad transverse medial band across entire width from suture towards lateral margin, c) subtriangular patch at apex and d) band along lateral margin confluent with basal and medial bands and subtriangular apical patch. Apical declivity along suture without scales, but with sparse golden-coloured suberect short hairs which continue and become denser towards apex. Legs with moderately clavate femora. Femora covered with recumbent brown hairs. Tibiae weakly serrate along inner margin with suberect, brown bristles along inner margin and subrecumbent brown hairs along outer margin. Fore and mid-tibiae bearing mucro at apex. All tarsomeres densely pubescent dorsally. Coxa with light-coloured recumbent hairs. Mesoventrite with sparse light-coloured, recumbent hairs. Metaventrite and ventrite I moderately depressed on disc, with light-coloured, adpressed hairs and yellow ochre, round scales towards lateral margins. Ventrites II–IV with sparse light-coloured, adpressed hairs. Ventrite V very weakly convex, densely punctured and with dense, long, light-brown adpressed hairs.

**Figure 2.**
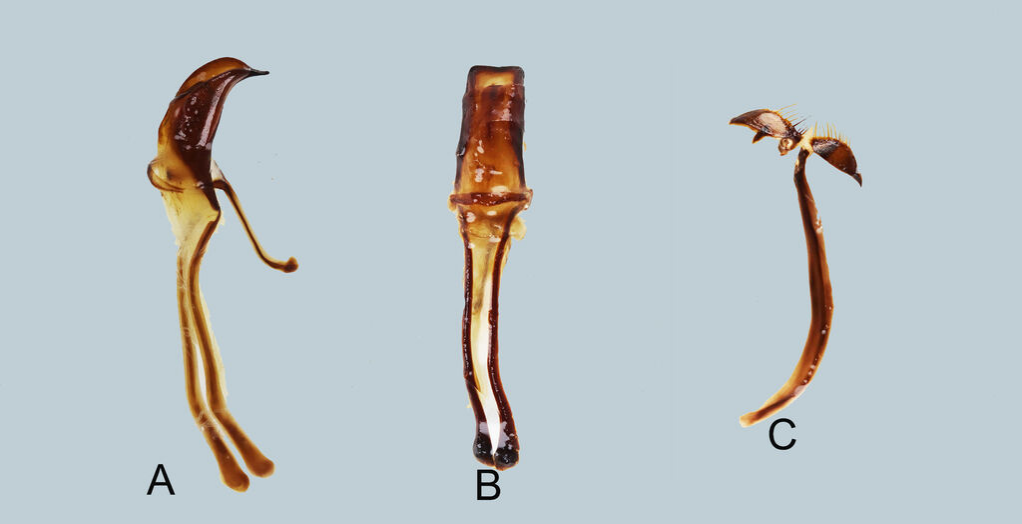
Male genitalia of *Metapocyrtusjumawani* **A** aedeagus in lateral view; **B** idem in dorsal view; **C** sternite IX in dorsal view.

**Male genitalia** as shown in Fig. 2A–C. Aedeagal body short and stout and bent towards the apex (in lateral view), apicad slightly subangulate, apex acute; aedeagal apodemes twice longer than aedeagal body.

Female. Dimensions (in mm): N = 4. LB 8.5–9.5 (â: 8.75), LR 1.5–1.8 (â: 1.58), WR 1.2, LP 2.8–3.0 (â: 2.85), WP 2.9–3.0 (â: 2.93), LE 5.7–6.5 (â: 5.9), WE 4.0–4.5 (â: 4.13).

Females differ from males by the following characters: a) pronotum slightly longer than wide in females (LP/WP 0.96-1.0) than in males, b) elytra slightly longer and wider (LE/WE 1.43–1.44, WE/WP 1.38–1.5, LE/LP 2.03–2.17) than in males, c) elytral apex more pointed and with denser hairs at apex and d) posterior elytral marking imperfectly subtriangular. Otherwise, females similar to males.

#### Diagnosis

*Metapocyrtusjumawani* sp. nov. is related to *Metapocyrtustagabawa* Cabras, Bollino & Medina, 2020 from Toril, Davao City and Wao, Lanao del Sur and *Metapocyrtuslatifasciatus* Cabras, Bollino & Medina, 2020 from Cotabato, Sarangani and Davao del Sur, but differs on the broader pronotal and elytral markings and the shape of the aedeagus. *M.jumawani* sp. nov. differs from *M.tagabawa* for having broader transverse pronotal mark and basal and medial transverse bands, as well as banded triangular marks at the apical third of the elytra; whereas it differs from *M.latifasciatus* by having thick transverse basal and medial bands in the elytra instead of one full broad basal band covering almost half of each elytron. At the moment, as the real validity and repartition amongst *Metapocyrtus* subgenera requires a complete revision, thus we avoided subgeneric assignment for this species.

#### Etymology

The specific epithet is named after Kim Jumawan (Philippines) for his camaraderie and contribution in helping collect the species described herein.

#### Distribution

*Metapocyrtusjumawani* sp. nov. is known, so far, only from the Province of Davao de Oro.

### Metapocyrtus (Metapocyrtus) ged

Cabras & Medina, 2021 sp. n.

26AFAEE6-25E9-5085-BCAD-C1FEAC3FE2E9

urn:lsid:zoobank.org:act:ef162a92-14a3-4bc0-8b8d-2d167b56307b

#### Materials

- **Type status:** Holotype. **Occurrence:** recordedBy: Local collector; individualCount: 1; sex: male; lifeStage: adult; preparations: card-mounted; disposition: in collection; **Taxon:** scientificName: *Metapocyrtusged*; kingdom: Animalia; phylum: Arthropoda; class: Insecta; order: Coleoptera; family: Curculionidae; genus: Metapocyrtus; specificEpithet: *ged*; taxonRank: species; scientificNameAuthorship: Cabras & Medina, 2021; nomenclaturalCode: ICZN; **Location:** continent: Asia; islandGroup: Mindanao; country: Philippines; countryCode: PHL; stateProvince: Davao del Sur; municipality: Davao City; locality: Toril; **Identification:** identifiedBy: AA Cabras; **Event:** samplingProtocol: beating sheet; year: 2019; month: May; habitat: old growth secondary forest; **Record Level:** institutionID: UM; collectionID: UM-CRC
- **Type status:** Paratype. **Occurrence:** recordedBy: Local collector; individualCount: 5; sex: male; lifeStage: adult; preparations: card-mounted; disposition: in collection; **Taxon:** scientificName: *Metapocyrtusged*; kingdom: Animalia; phylum: Arthropoda; class: Insecta; order: Coleoptera; family: Curculionidae; genus: Metapocyrtus; specificEpithet: *ged*; taxonRank: species; scientificNameAuthorship: Cabras & Medina, 2021; nomenclaturalCode: ICZN; **Location:** continent: Asia; islandGroup: Mindanao; country: Philippines; countryCode: PHL; stateProvince: Davao del Sur; municipality: Davao City; locality: Carmen; **Identification:** identifiedBy: AA Cabras; **Event:** samplingProtocol: hand picking; year: 2019; month: July; habitat: old growth secondary forest; **Record Level:** institutionID: UM; collectionID: UM-CRC
- **Type status:** Paratype. **Occurrence:** recordedBy: Local collector; individualCount: 2; sex: female; lifeStage: adult; preparations: card-mounted; disposition: in collection; **Taxon:** scientificName: *Metapocyrtusged*; kingdom: Animalia; phylum: Arthropoda; class: Insecta; order: Coleoptera; family: Curculionidae; genus: Metapocyrtus; specificEpithet: *ged*; taxonRank: species; scientificNameAuthorship: Cabras & Medina, 2021; nomenclaturalCode: ICZN; **Location:** continent: Asia; islandGroup: Mindanao; country: Philippines; countryCode: PHL; stateProvince: Davao del Sur; municipality: Davao City; locality: Toril; **Identification:** identifiedBy: AA Cabras; **Event:** samplingProtocol: beating sheet; year: 2019; month: May; habitat: old growth secondary forest; **Record Level:** institutionID: UM; collectionID: UM-CRC
- **Type status:** Paratype. **Occurrence:** recordedBy: Local collector; individualCount: 1; sex: female; lifeStage: adult; **Taxon:** scientificName: *Metapocyrtusged*; kingdom: Animalia; phylum: Arthropoda; class: Insecta; order: Coleoptera; family: Curculionidae; genus: Metapocyrtus; specificEpithet: *ged*; taxonRank: species; scientificNameAuthorship: Cabras & Medina, 2021; nomenclaturalCode: ICZN; **Location:** continent: Asia; islandGroup: Mindanao; country: Philippines; countryCode: PHL; stateProvince: Davao del Sur; municipality: Davao City; locality: Carmen; **Identification:** identifiedBy: AA Cabras; **Event:** samplingProtocol: hand picking; year: 2019; month: July; habitat: old growth secondary forest; **Record Level:** institutionID: UM; collectionID: UM-CRC

#### Description

**Male.** Dimensions (in mm): N = 2. LB 7.8–8.1 (holotype 7.8, â: 7.95), LR 1.2–1.3 (1.3, â: 7.95), WR 1.1–1.2 (1.1, â: 1.05), LP 3.0–3.1 (3.0, â: 3.05), WP 2.8–2.9 (2.8, â: 2.85), LE 4.8–5.0 (4.8, â: 4.9), WE 3.5–3.7 (3.5, â: 4.9).

**Figure 3.**
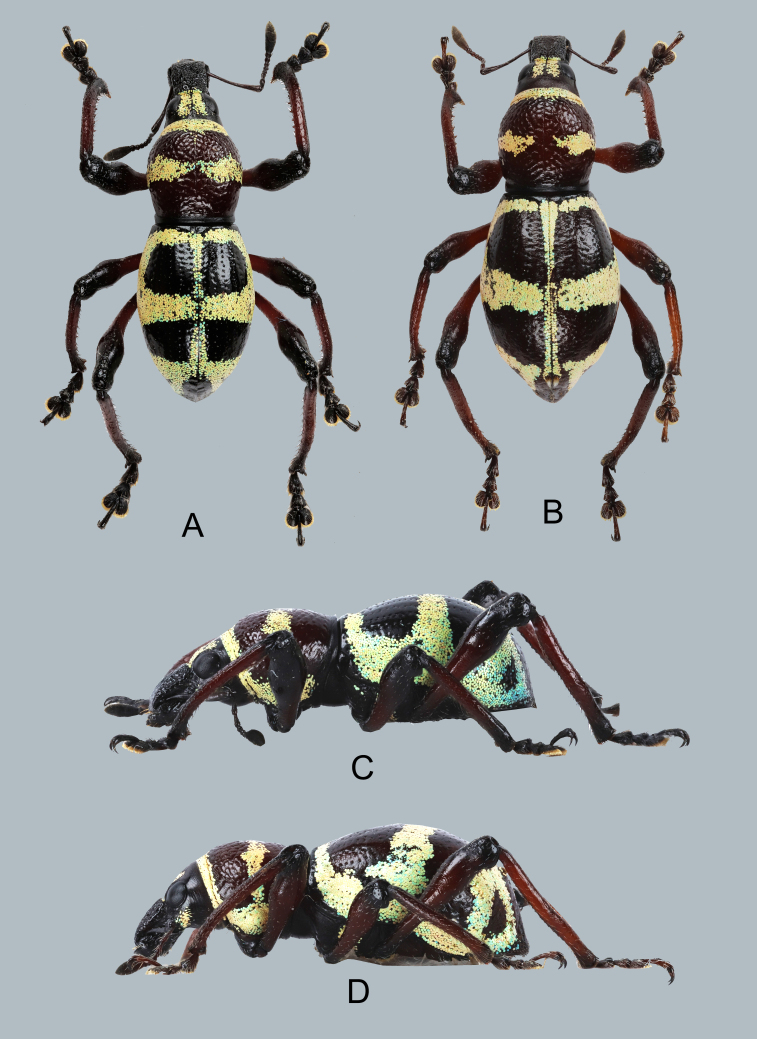
Metapocyrtus (Metapocyrtus) ged sp. nov.: **A** male holotype, dorsal view; **B** female, dorsal view; **C** male, lateral view; **D** female, lateral view.

Habitus as shown in Fig. 3A–D.

Integument of elytra black; integument of pronotum, head, scape and legs dark ferruginous; funicle, tarsi, rostrum black. Body surface, rostrum and head weakly lustrous and underside subopaque.

Body subglabrous. Head subglabrous, sparsely, minutely pubescent; lateroventral parts with yellow ochre and turquoise round and elliptic scales interspersed with light-coloured recumbent piliform scales, forehead between eyes slightly depressed, covered with metallic, yellow ochre and turquoise round and elliptic scales; median groove indistinct. Ros­trum strongly rugose especially in basal half, sparsely, minutely pubescent, slightly longer than wide (LR/WR 1.18), dorsum bearing V-shape ridge and minute, light-coloured hairs; lateral surface with recumbent light-coloured hairs and long, light-brown hairs at the anterolateral mar­gin; transverse basal groove distinct, beset with sparse, light yellow ochre and torquiose round scales; dorsal surface moderately convex. Eyes medium-sized and feebly convex. Antenna clavate, scape as long as funicle, moderately covered with fine, light-coloured hairs. Funicular antennomeres I and II of the same length, nearly three times longer than wide; antennomeres III–VII as long as wide; club subovoid, nearly three times longer than wide. Prothorax globular, slightly longer than wide (LP/WP 1.07), weakly granulated with sparse pubescence, widest at mid-length, moderately convex, with a faint groove along mid-line and with the following scaly markings of metallic yellow ochre, turquoise and light blue, round scales: a) thin band approximately 1/6 length of pronotum along anterior margin, b) thin transverse band at mid-length, sometimes interrupted at centre and c) lateroventral stripe before the coxa confluent with the anterior and median bands. Elytra subelliptical (LE/WE 1.37), slightly wider and twice longer than prothorax (WE/WP 1.25, LE/LP 1.6), subglabrous, striate-punctate, weakly convex, with very minute and sparse light-coloured pubescence. Elytra with the following scaly markings of yellow ochre and turquoise, round scales: a) basal transverse band from suture towards the lateral sides, b) median transverse band, c) subtriangular patch on apical third, d) thick band along lateral margin confluent with basal band, median band and triangular patch at apical third and e) faint stripe along suture. The elytral marks seem to create four squares on the dorsum of the elytra. Apex of elytra with erect bluish piliform scales and light-coloured hairs. Legs with strongly clavate femora. Femora dark ferruginous, but black towards apical half, covered with bluish recumbent piliform scales. Tibiae dark ferruginous, but black towards apex, covered with black piliform scales towards outer surface and black erect hairs on inner margin; serrate along inner margin. Fore and mid-tibiae bearing mucro at apex. Tarsomeres covered with pubescence dorsally. Coxae with light-coloured to greenish recumbent piliform scales. Mesoventrite with sparse and minute light-coloured hairs. Metaventrite with light-coloured suberect hairs and pale yellow to turquoise, round scales at lateral sides. Ventrite I faintly depressed on disc with light-coloured, suberect hairs and sparse light-coloured piliform scales towards sides. Ventrite II with short, light-coloured, suberect hairs and sparse light-coloured piliform scales towards sides. Ventrites III–IV with sparse, light-coloured, short hairs. Ventrite V flattened, apical half finely densely punctured with suberect hairs.

**Figure 4.**
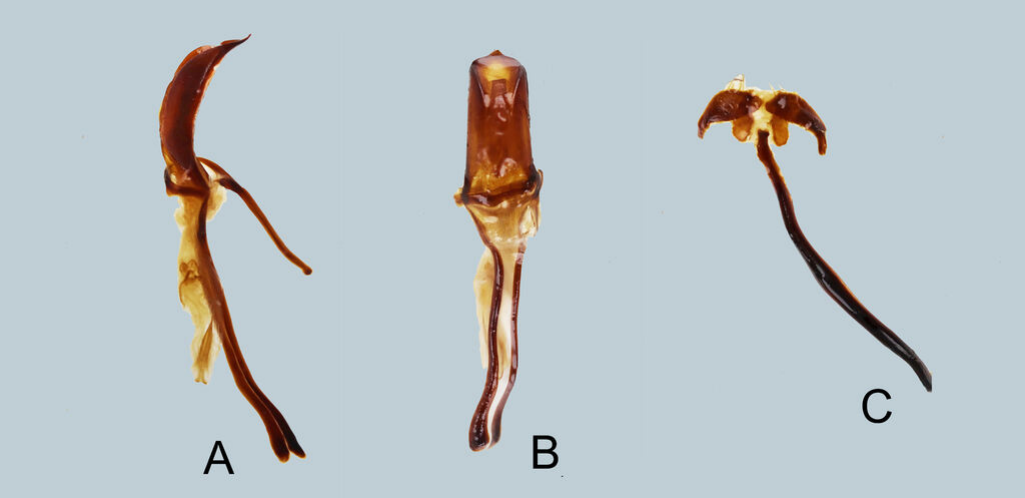
Male genitalia of *Metapocyrtusged* **A** aedeagus in lateral view; **B** idem in dorsal view; **C** sternite IX in dorsal view.

**Male genitalia** as shown in Fig. 4A-C. Aedeagal body short and slender and apicad bluntly produced with acute apex; aedeagal apodemes twice longer than aedeagal body.

Female. Dimensions (in mm): N = 2. LB 9.0–9.3 (â: 9.2), LR 1.0–1.1 (â: 1.05), WR 1.2–1.4 (â: 1.3), LP 3.0–3.1 (â: 3.05), WP 3.0–3.1 (â: 3.05), LE 6.0–6.2 (â: 6.1), WE 4.0–4.2 (â: 4.1).

Females differ from males by the following characters: a) pronotum more convex, b) elytra obovate (LE/WE 1.47–1.62), longer and wider (WE/WP 1.33–1.35, LE/LP 2.0) than in males, c) apex of elytral declivity with tuft of bristles and e) ventrite I flattened or slightly convex on disc. Otherwise, females are similar to males.

#### Diagnosis

*Metapocyrtusged* sp. nov. belongs to the subgenusMetapocyrtus s.s. for its rostrum with V-shape ridge and rounded dorsolateral edges, as well as elytra laterally elliptical or dorsally ovate. The species is distinct from the rest of *Metapocyrtus* s.s. by its unique elytral ornamentation.

#### Etymology

The specific epithet *ged* is after the acronym of Gloria E. Detoya, Chief Operating Officer/Quality Management Representative of the University of Mindanao for her unwavering support to the Coleoptera Research Center.

#### Distribution

The new species is, so far, known only in the Province of Davao del Sur.

### Metapocyrtus (Metapocyrtus) flavomaculata sp. n.

E7836DF0-F336-53BA-B427-4AF672C650C7

urn:lsid:zoobank.org:act:54054f64-a5d5-457e-b40b-37165b3588f4

#### Materials

- **Type status:** Holotype. **Occurrence:** recordedBy: Local collector; individualCount: 1; sex: male; lifeStage: adult; preparations: card-mounted; disposition: in collection; **Taxon:** scientificName: *Metapocyrtusflavomaculata*; kingdom: Animalia; phylum: Arthropoda; class: Insecta; order: Coleoptera; family: Curculionidae; genus: Metapocyrtus; specificEpithet: *flavomaculata*; taxonRank: species; scientificNameAuthorship: Cabras & Medina, 2021; nomenclaturalCode: ICZN; **Location:** continent: Asia; islandGroup: Mindanao; country: Philippines; countryCode: PHL; stateProvince: Davao del Sur; municipality: Davao City; locality: Lamanan; **Identification:** identifiedBy: AA Cabras; **Event:** samplingProtocol: handpicking; year: 2018; month: July; habitat: old growth secondary forest; **Record Level:** institutionID: UM; collectionID: UM-CRC
- **Type status:** Paratype. **Occurrence:** recordedBy: Local collector; individualCount: 5; sex: female; lifeStage: adult; preparations: card-mounted; disposition: in collection; **Taxon:** scientificName: *Metapocyrtusflavomaculata*; kingdom: Animalia; phylum: Arthropoda; class: Insecta; order: Coleoptera; family: Curculionidae; genus: Metapocyrtus; specificEpithet: *flavomaculata*; taxonRank: species; scientificNameAuthorship: Cabras & Medina, 2021; nomenclaturalCode: ICZN; **Location:** continent: Asia; islandGroup: Mindanao; country: Philippines; countryCode: PHL; stateProvince: Davao del Sur; municipality: Davao City; locality: Lamanan; **Identification:** identifiedBy: AA Cabras; **Event:** samplingProtocol: handpicking; year: 2018; month: July; habitat: old growth secondary forest; **Record Level:** institutionID: UM; collectionID: UM-CRC

#### Description

**Male.** Dimensions (in mm): N = 2. LB 6.8–7.1 (holotype 10.9, â: 10.75), LR 1.0–1.1 (1.0, â: 1.05), WR 1.0–1.1 (1.0, â: 1.05), LP 2.0–2.1 (2.0, â: 2.05), WP 2.0–2.1 (2.0, â: 2.05), LE 4.8–5.0 (4.8, â: 4.9), WE 3.0–3.2 (3.0, â: 3.1).

**Figure 5.**
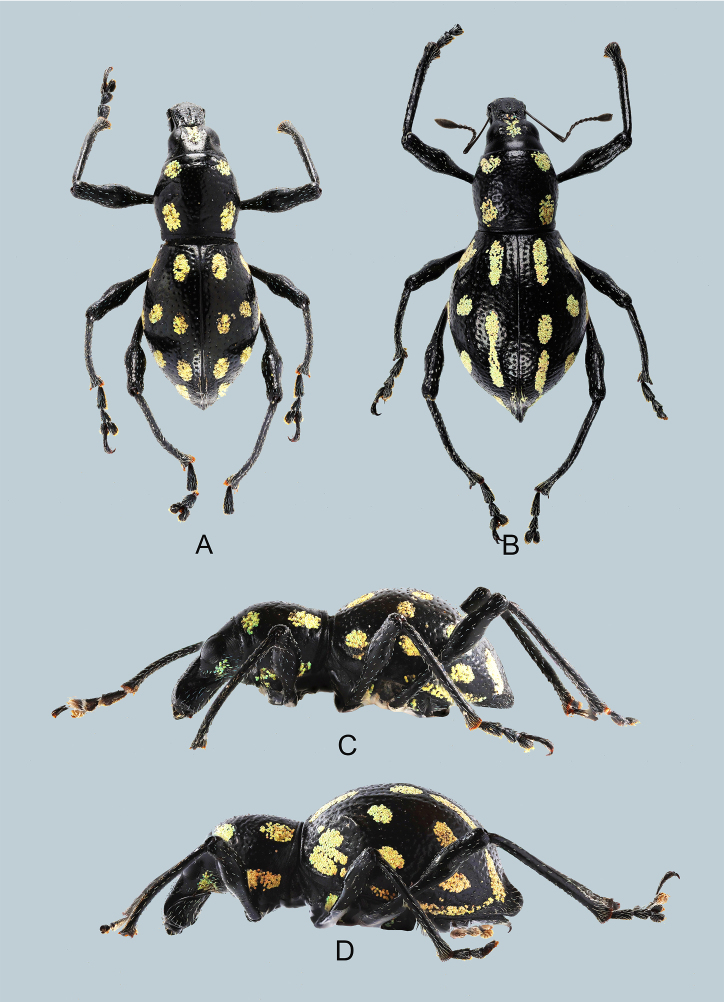
*Metapocyrtusflavomaculata* sp. nov.: **A** male holotype, dorsal view; **B** female, dorsal view; **C** male, lateral view; **D** female, lateral view.

Habitus as shown in Fig. 5B and D.

Integument black. Body surface, rostrum, head and underside with weak lustre.

Body subglabrous. Head subglabrous, sparsely, very minutely pubescent; forehead between eyes slightly depressed and covered with metallic, light-yellow ochre, round scales; lateral parts with light-yellow ochre, round scales behind eye interspersed with metallic yellow-green, hairlike, elliptical scales; median groove distinct. Ros­trum sparsely, minutely pubescent, slightly longer than wide (LR/WR 1.33), dorsum bearing minute, light-coloured recumbent hairs; lateral surface with metallic, yellow-green hair-like scales and long, light-coloured hairs towards anterolateral mar­gin; transverse basal groove distinct; dorsal surface weakly convex with faint V-shaped ridge. Eyes medium-sized and moderately convex. Antenna moderately clavate, scape slightly shorter than funicle, moderately covered with fine, light-coloured hairs. Funicular antennomeres I and II almost of the same length, nearly three times longer than wide; antennomeres III–VII slightly longer than wide; club subovoid, nearly three times longer than wide. Prothorax subglobular, as long as wide (LP/WP 1.0), faintly punctured with sparse minute hairs, widest at mid-length, weakly convex and with the following scaly markings of metallic light yellow ochre and pale green, round scales: a) two medium-sized spots on basal parts, b) two medium-sized spots along apical margin and c) lateroventral stripe before the coxa. Elytra strongly ovate (LE/WE 1.6), wider and longer than prothorax (WE/WP 1.5, LE/LP 2.4), black, subglabrous, moderately convex, coarsely punctured. Each elytron with the following markings of metallic light-yellow ochre and pale green round scales: a) two sub-basal spots, b) three spots slightly before mid-length, c) three spots along apical third and d) one short apical stripe. Suture along apical declivity with light-coloured piliform scales becoming denser towards apex. Legs with strongly clavate femora. Femora covered with recumbent light-bluish hair-like scales. Tibiae covered with suberect, light-coloured bristles along inner margin and light-coloured recumbent hairs on outer margin; weakly serrate along inner edge. Fore tibiae bearing mucro at apex. Tarsomeres covered with pubescence dorsally. Coxae with light-coloured suberect hair-like scales. Mesoventrite covered with suberect light-coloured hairs. Metaventrite and ventrite I slightly depressed with light-coloured, adpressed bristles and light-yellow ochre, round scales at lateral sides. Ventrites II–V with light-coloured, adpressed bristles. Ventrite V flattened, smooth with dense, recumbent yellow-ochre hairlike-scales.

**Figure 6.**
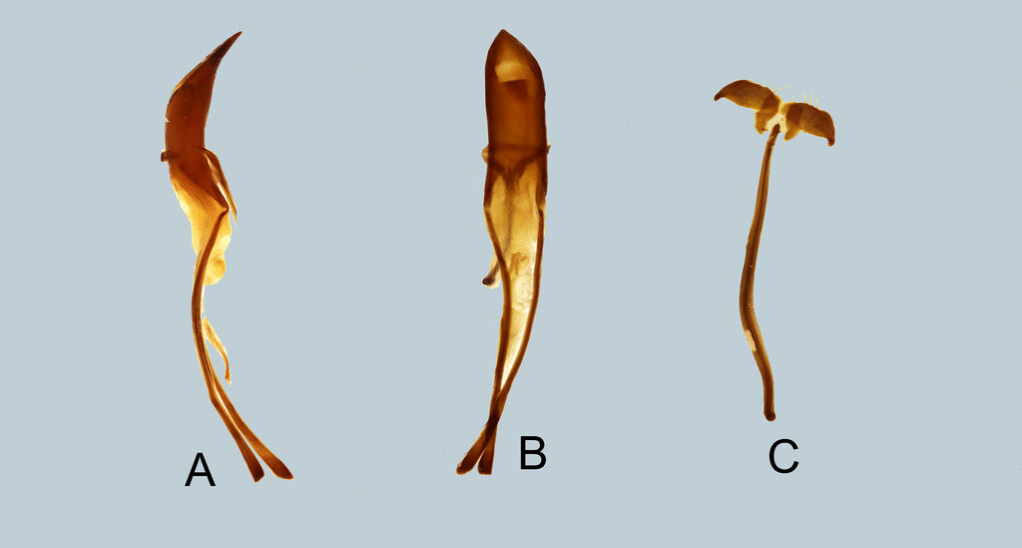
*Metapocyrtusflavomaculata* **A** aedeagus in lateral view; **B** idem in dorsal view; **C** sternite IX in dorsal view.

**Male genitalia** as shown in Fig. 6A–C. Aedeagal body short and slender and apicad sharply produced with acute apex; aedeagal apodemes 2.5 longer than aedeagal body.

Female. Dimensions (in mm): N = 6. LB 7.6–8.5 (â: 8.2), LR 1.0–1.2 (â: 1.13), WR 0.8–1.0 (â: 0.93), LP 2.1–2.5 (â: 2.37), WP 2.4–2.8 (â: 2.67), LE 5.5–6.0 (â: 5.83), WE 4.0–4.2 (â: 4.13).

Females differ from males by the following characters: a) pronotum slightly shorter than wide in males (LP/WP 0.88–0.89), b) pronotum subtruncate, c) elytra longer and wider (LE/WE 1.38–1.43, WE/WP 1.5–1.67, LE/LP 2.4–2.62) than in males and with oblique faint ridge on dorsolateral surface before mid-length, d) presence of tuft of piliform scales on elytral declivity and e) elytral apex with triangular projection. Otherwise mentioned, females similar to males.

#### Diagnosis

Metapocyrtus (Metapocyrtus) flavomaculata sp. nov. differs from all other species of the subgenus for its unique elytral ornamentation consisting of yellow ochre spots. It bears some superficial resemblance to Metapocyrtus (Metapocyrtus) worcesteri Schultze, 1925 from Zamboanga Province, but can be easily distinguished by the shape of the rostrum and pronotum, the presence of four spots in the pronotum (two at basal parts and two along apical margin), compared to the two spots at mid-length of M. (M.) worcesteri. The presence of an oblique ridge on the dorsolateral surface before mid-length in the elytra of females of Metapocyrtus (Metapocyrtus) flavomaculata sp. nov. also distinguishes it.

#### Etymology

Etymology. Latin, flavus = yellow; maculata = spotted. The Latin name refers to the yellow spots on the elytra.

#### Distribution

The new species is known, so far, in Davao City and Bukidnon.

### Metapocyrtus (Metapocyrtus) pseudahirakui

Cabras & Medina, 2021 sp. n.

24BB00C7-FBA3-5B6A-ABE4-2AEF39242D36

urn:lsid:zoobank.org:act:7f58a8b0-4f64-4f23-a798-2c3d377e9c61

#### Materials

- **Type status:** Holotype. **Occurrence:** recordedBy: Local collector; individualCount: 1; sex: female; lifeStage: adult; preparations: card-mounted; disposition: in collection; **Taxon:** scientificName: *Metapocyrtuspseudahirakui*; kingdom: Animalia; phylum: Arthropoda; class: Insecta; order: Coleoptera; family: Curculionidae; genus: Metapocyrtus; specificEpithet: *pseudahirakui*; taxonRank: species; scientificNameAuthorship: Cabras & Medina, 2021; nomenclaturalCode: ICZN; **Location:** continent: Asia; islandGroup: Mindanao; country: Philippines; countryCode: PHL; stateProvince: Bukidnon; municipality: Lantapan; **Identification:** identifiedBy: AA Cabras; **Event:** samplingProtocol: Beating sheet; year: 2018; month: July; habitat: old growth primary forest; **Record Level:** institutionID: UM; collectionID: UM-CRC

#### Description

**Female.** Dimensions (in mm): LB 10.2, LR 1.0, WR 1.0, LP 3.2, WP 3.2, LE 7.0, WE 4.8, N = 1.

**Figure 7.**
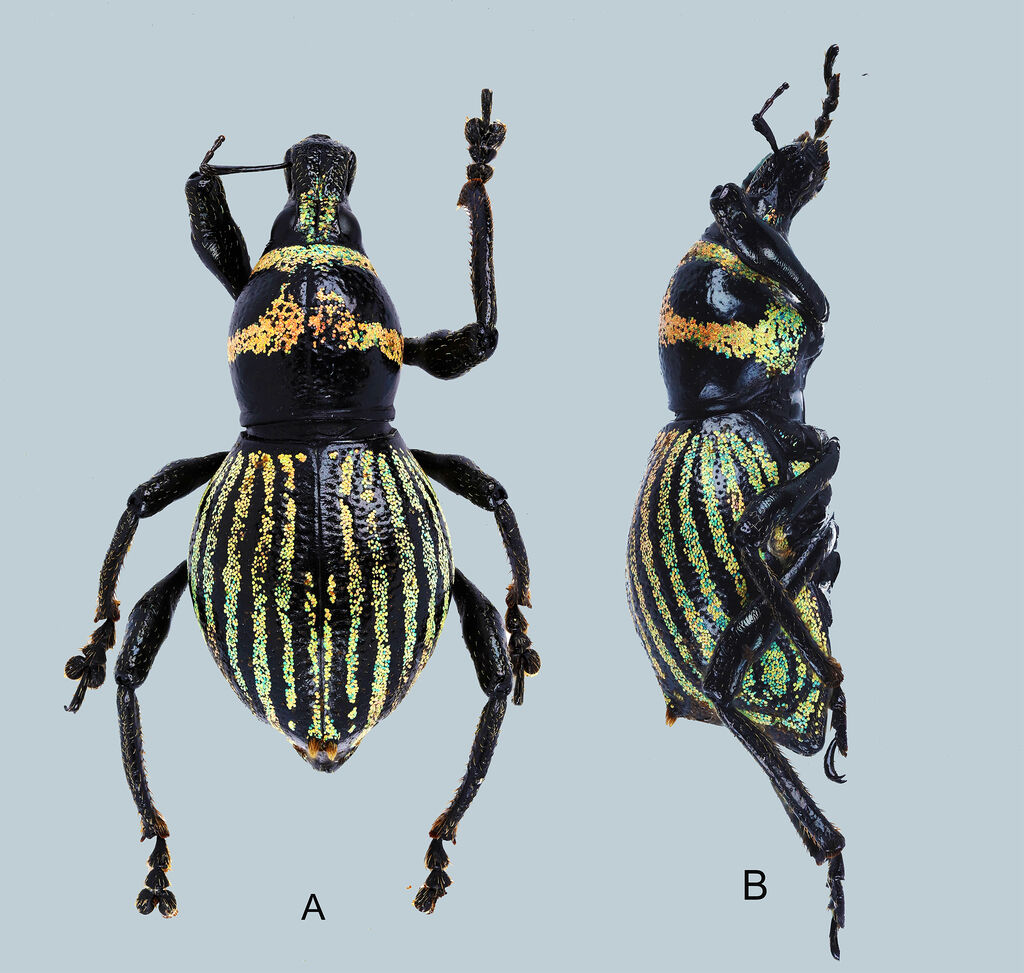
Metapocyrtus (Metapocyrtus) pseudahirakui sp. nov., female holotype: **A** dorsal view; **B** lateral view.

Habitus as shown in Fig. 7.

Integument black. Body surface, rostrum, head and underside with weak lustre.

Body subglabrous. Head subglabrous, sparsely, minutely pubescent; forehead between eyes flattish, covered with metallic, golden-yellow and round scales; median groove barely distinct; lateral parts behind eyes with golden-yellow and pale green round scales interspersed with hair-like scales of similar colours. Ros­trum sparsely, minutely pubescent, slightly longer than wide (LR/WR 1.33), dorsum and lateral surface bearing minute, recumbent, yellowish hairlike-scales and anterolateral mar­gin with longer, light-brown hairs; transverse basal groove distinct; basal half with pronounced V-shaped ridge beset with metallic golden-yellow, round scales. Eyes medium-sized and feebly convex. Antenna moderately covered with fine, light-coloured hairs. Funicular antennomeres I and II almost of the same length, nearly three times longer than wide. Prothorax subglobular, as long as wide (LP/WP 1.0), mostly punctured, coarsely rugose and punctured especially along middle of dorsum, weakly convex, with distinct mid-line groove. Pronotum with the following scaly markings of metallic golden-yellow and pale green, round scales: a) thin transverse band approximately one seventh of the length of pronotum at anterior margin, b) transverse band across entire width at mid-length slightly thicker than anterior band, nearly confluent with anterior band at middle of dorsum and c) lateroventral stripe before coxa, confluent with anterior and median bands. Elytra short ovate (LE/WE 1.46), wider and longer than prothorax (WE/WP 1.5, LE/LP 2.19), black, subglabrous, strongly convex; coarsely punctured with very minute and sparse light-coloured, short seta; slightly flattish on basal half of dorsum and with steep apical declivity. Elytra with eight longitudinal scaly stripes of metallic golden-yellow and pale green, round scales beginning shortly from anterior margin and extending towards apex and one longitudinal parasutural stripe from before mid-length towards apical declivity. Longitudinal stripe at striae II extending from anterior margin to apical declivity; stripe at striae II extending from anterior margin to apex of elytra, confluent with stripe at lateral margin; stripe at striae III–VII confluent at apical quarter. Base of apical declivity with brownish tuft of hairs on each elytron. Legs with strongly clavate femora. Femora covered with recumbent light-bluish hair-like scales. Tibiae covered with suberect, light-coloured bristles along inner margin and light coloured recumbent hairs on outer margin; weakly serrate along inner edge. Fore and mid-tibiae bearing mucro at apex. Tarsomeres covered with pubescence dorsally. Coxae with recumbent light-coloured hair-like scales. Mesoventrite covered with metallic and greenish, adpressed hair-like scales. Metaventrite slightly depressed on disc with subrecumbent hair-like scales and light-yellow ochre and pale green round scales at lateral sides. Ventrite I depressed on disc with light-coloured, adpressed hairs. Ventrites II–V with sparse, light-coloured, short hairs. Ventrite V flattened, finely densely punctured with short hairs.

Males unknown.

#### Diagnosis

Metapocyrtus (M.) pseudahirakui sp. nov. differs from all other species of the subgenus for its unique elytral ornamentation consisting of golden-yellow and pale green longitudinal stripes along the elytral striae. It superficially looks similar to M. (Orthocyrtus) hirakui Cabras, Medina & Bollino, 2021 due to mimicry reasons, but can be distinguished by its rostrum, punctures of the pronotum and the tuft of brownish hairs at the base of the apical declivity. M. (M.) pseudahirakui sp. nov. has rostrum with V-shape ridge and rounded dorsolateral edges, whereas M. (O.) hirakui has a rostrum which is dorsally straight, mostly in a plane with front and, at base, the sides are rectangularly declined.

#### Etymology

Etymology. The specific epithet pertains to its uncanny resemblance to M. (O.) hirakui.

#### Distribution

Metapocyrtus (M.) pseudahirakui sp. nov. is known, so far, in Bukidnon.

## Discussion

**Notes on the ecology and mimicry of the new *Metapocyrtus* spp.**

The type locality of *M.jumawani* sp. nov. is in Maragusan, Davao de Oro. Davao de Oro (formerly Compostela Valley or COMVAL) Province in Davao Region and is part of the Eastern Mindanao Biological Corridor (EMBC). It is a long stretch of mountain ecosystems bordering the eastern seaboard of Mindanao. The mountain ecosystem of Davao de Oro, including Cagan Valley, is situated in the east side of Mt. Candalaga range. The biotype is composed of old growth secondary forests, mostly remnants of the logging concessions in the late 1950s. Due to its rugged terrain and inaccessibility for most villagers in the nearby downtown, its forests have remained intact and undisturbed for decades, including its riparian systems, making it an ideal habitat for members of the tribe Pachyrhynchini. The new species was collected on the leaves of *Elatostema* sp. (Urticaceae).

**Figure 8.**
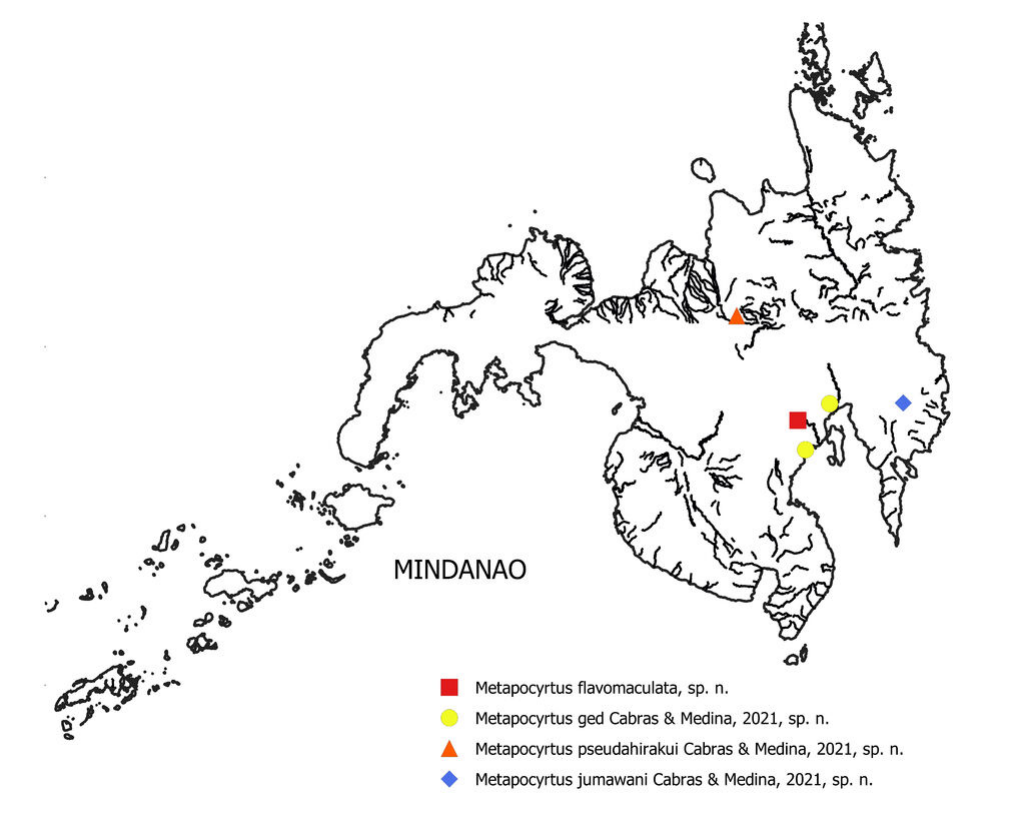
Geographical distribution map of *Metapocyrtus* spp. on Mindanao Island

As for M. (M.) ged sp. nov. and M. (M.) flavomaculata sp. nov., both species were collected in Davao City. M. (M.) ged sp. nov. was collected in the secondary forests of Catigan, Toril (Fig. 8) using a modified beating sheet method. This modified method involves putting a 2 x 4 white cloth at the base of trees and shrubs and shaking the trees and shrubs vigorously so the non-flying insects will fall to the cloth. M. (M.) flavomaculata sp. nov., on the other hand, was collected in the montane forests bordering Lamanan and Marilog, at an elevation of approximately 1000–1200 m a.s.l. The area where the specimens were collected is a remnant of an old growth forest and the rocks were mostly covered by moss. The new species was collected in a shady area and mostly on wild *Impatiens* sp. (Balsaminaceae) plants growing on huge mossy rocks. M. (M.) flavomaculata sp. nov. was also observed in Lantapan, Bukidnon which extends the range of the distribution of the species. The northern part of Davao City, such as Calinan, Toril, Lamanan and Marilog, share some common species with Bukidnon, such as the recently-described *M.kitangladensis* Cabras, Medina & Zhang, 2019 which was found abundant in Bukidnon and was also observed in Marilog and Carmen in Davao City. Lantapan Bukidnon is also the type locality of M. (M.) pseudahirakui sp. nov. The species was collected alongside M. (O.) hirakui Cabras, Medina & Bollino, 2021 and *Pachyrhynchustikoi* Rukmane, 2016, which were very abundant in the area. At first, it can easily be mistaken for M. (O.) hirakui until further microscopic examination.

**Figure 9.**
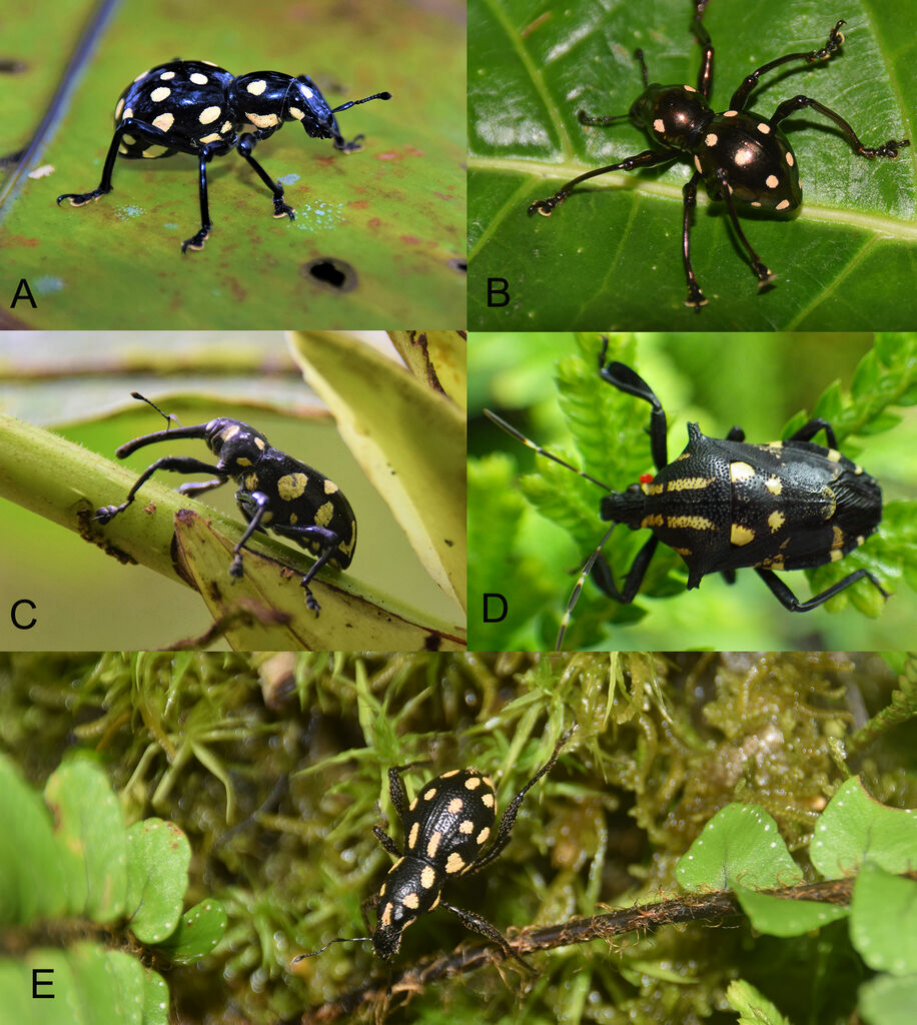
Mimicry of *Metapocyrtusflavomaculata* sp. nov. **A** *Pachyrhynchussulphureomaculatus*; **B** *Pachyrhynchuserichsoni*; **C** *Alcidodes* sp. (Curculionidae, Alcidinae); **D** *Platynopusmelanoleucus* (Westwood, 1837) (Pentatomidae); **E** . *Metapocyrtusflavomaculata* sp. nov.

*Metapocyrtuspseudahirakui* sp. nov. is a classic example of interspecific mimicry within the genus *Metapocyrtus*, which was mentioned by Schultze (1925). Mimicry within the tribe Pachyrhynchini is quite common, especially between *Pachyrhynchus* and *Metapocyrtus* (*Schultze 1925*). Amongst the four species described here, three are involved in mimicry complexes. *M.jumawani* sp. nov. is involved in a large and complex mimicry with several species of *Metapocyrtus*, as well as *Pachyrhynchuskraslavae* Rukmane & Barševskis 2016 and *Pachyrrhynchusmiltoni* Cabras & Rukmane, 2016 in Davao de Oro. *Metapocyrtuspseudahirakui* sp. nov. is in a mimicry complex with M. (O.) hirakui Cabras, Medina & Bollino, 2020, *Pachyrhynchustikoi* Rukmane, 2016, *Doliopsdauvapilsi* Barševskis, 2014 and *Polycatusmimicus* Bramanti, Bramanti, & Rukmane, 2020. Another remarkable mimicry complex is the one observed with M. (M.) flavomaculata sp. nov., wherein five species from the order Coleoptera and one species of Hemiptera were documented sharing the same ornamentations of yellow spots on their bodies within the same vicinity. Amongst the members of this mimicry complex are *Pachyrhynchussulphureomaculatus*, *Pachyrhynchuserichsoni*, *Alcidodes* sp. and the true bug *Platynopusmelanoleucus* (Westwood, 1837) (Pentatomidae) (Fig. 9) . The involvement of crickets and true bugs in the mimicry complex of the tribe Pachyrhynchini was already noted long ago by Wallace (1889). Mimicry amongst the tribe Pachyrhynchini was first noted by early naturalists who worked in Southeast Asia such as Wallace and Schultze, who noticed sympatric species of Pachyrhynchini sharing the same integument colours and elytral patterns. The tribe Pachyrhynchini are taking advantage of their colouration as aposematic signals in deterring predators, exploiting predators’ visual system and making them unpalatable to predators (Tseng et al. 2014). This defence mechanism was utilised by other members of weevils, such as *Macrocyrtus*, *Celebia*, *Alcidodes*, *Polycatus*, *Eupyrgops*, *Neopyrgops*, *Coptorhynchus* and even the longhorned beetle *Doliops* by copying the patterns and colourations of the tribe Pachyrhynchini.

## Supplementary Material

XML Treatment for Metapocyrtus jumawani

XML Treatment for Metapocyrtus (Metapocyrtus) ged

XML Treatment for Metapocyrtus (Metapocyrtus) flavomaculata

XML Treatment for Metapocyrtus (Metapocyrtus) pseudahirakui
